# Monolithically-Integrated TE-mode 1D Silicon-on-Insulator Isolators using Seedlayer-Free Garnet

**DOI:** 10.1038/s41598-017-06043-z

**Published:** 2017-07-19

**Authors:** Cui Zhang, Prabesh Dulal, Bethanie J. H. Stadler, David C. Hutchings

**Affiliations:** 1 School of Engineering, University of Glasgow, Glasgow, G12 8LS United Kingdom; 2 Chemical Engineering and Materials Science, University of Minnesota, Minneapolis, Minnesota 55455 United States; 3 Electrical and Computer Engineering, University of Minnesota, Minneapolis, Minnesota 55455 United States

## Abstract

The first experimental TE-mode silicon-on-insulator (SOI) isolators using Faraday Rotation are here realized to fill the ‘missing link’ in source-integrated near infrared photonic circuits. The isolators are simple 1D 2-element waveguides, where garnet claddings and longitudinal magnetic fields produce nonreciprocal mode conversion, the waveguide equivalent of Faraday Rotation (FR). Quasi-phase matched claddings are used to overcome the limitations of birefringence. Current experimental SOI isolators use nonreciprocal phase shift (NRPS) in interferometers or ring resonators, but to date NRPS requires TM-modes, so the TE-modes normally produced by integrated lasers cannot be isolated without many ancillary polarisation controls. The presented FR isolators are made via lithography and sputter deposition, which allows facile upscaling compared to the pulsed laser deposition or wafer bonding used in the fabrication of NRPS devices. Here, isolation ratios and losses of 11 dB and 4 dB were obtained, and future designs are identified capable of isolation ratios >30 dB with losses <6 dB.

## Introduction

Every critical line in a fiber optic system has a fiber-integrated nonreciprocal isolator to mitigate unwanted back reflections. However, in planar waveguide photonics, e.g. silicon-on-insulator (SOI), isolators and other devices that break time reversal symmetry are the ‘missing link’ that prevents low-noise source lasers from being integrated into the system^1, 2^. Part of the problem is that integrated photonic lasers normally emit TE-polarised light, which matches the favorable mode of most photonic waveguides. However, the passive nonreciprocal devices that have been experimentally verified using SOI waveguides to date generally only isolate TM-polarised light^1–8^. The functional components of these devices utilize nonreciprocal phase shift (NRPS), a phenomenon that acts only on TM modes when garnet top-claddings are used together with a transverse magnetic field. Although garnet side-claddings have been proposed for TE-mode devices using both Si and Si_x_N_y_ cores^9, 10^, these have yet to be realized on SOI platforms. Non-garnet alternatives that break time reversal symmetry are all active devices that require power to operate^11–15^.

In this report, we present the first experimental realization of passive TE-mode SOI-integrated isolators with a uniform longitudinal magnetisation causing Faraday Rotation (FR), or nonreciprocal polarisation conversion, the same phenomenon as that used in conventional optical isolators. These SOI FR isolators are similar in size to TM-mode NRPS Mach-Zehnder interferometers (MZI) and ring resonators. For example, the smallest NRPS MZI designs are 0.5 mm × 0.5 mm^4^, NRPS ring resonators are typically 0.3 mm × ~50 µm (width includes a linear waveguide next to a ring)^3^, and the FR waveguide devices presented here are 4 mm × ~1 µm. In fact, FR isolators are essentially 1D (all in a continuous line) rather than 2D (e.g., to incorporate interferometer branches and rings), and this 1D geometry will enable very high device densities.

**Figure 1:**
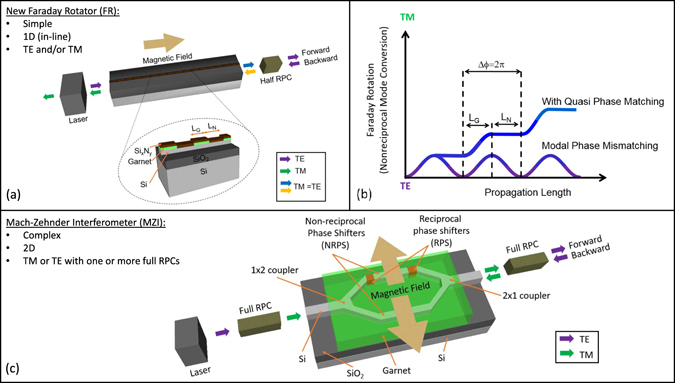
Comparison of a Faraday Rotation waveguide vs the simplest form of a Mach-Zehnder Interferometer (MZI), both as TE-mode isolators on Si-on-insulator (SOI) waveguides with garnet top claddings. (**a**) Schematic showing the components required for a Faraday Rotation waveguide isolator. The total isolator size is 4 mm × ~1 µm. (**b**) Schematic showing both the effects of phase velocity mismatch (birefringence) and the solution: quasi phase matching (QPM). QPM can be used to periodically suspend Faraday Rotation, or more specifically mode conversion, by spatially varying the cladding such that the period L_G_ + L_N_ corresponds to a modal phase offset of Δφ = 2π. (**c**) Schematic showing the components required for the simplest MZI-based isolator. A total size of 4 mm × 0.2 mm has been achieved using serpentine MZI^20^.

Unlike NRPS MZIs and ring resonators, FR isolators only require two elements for TE-mode operation: the Faraday Rotator (FR) and a half reciprocal polarisation converter (RPC), Fig. 1a. The RPC is used to convert the [TE = TM] output of the FR to full TE-mode before the light continues into the photonic integrated circuit. This is considered a half converter because the light is only required to convert from half TE to full TE, and in principle should be more straightforward to realise than a single-step full converter for mode-beating solutions on planar structures^16^. Although birefringence is a mismatch in the TE and TM modal phase velocities that limits the transfer of energy between the modes in the FR, quasi-phase matching (QPM) can be used to compensate for the mismatch and ensure a monotonic flow of energy between the modes^17^, Fig. 1b. QPM requires a spatial modulation in the mode-conversion process, for example, by avoiding FR where the phase relation has the opposite sign for growth of the desired mode component. This is implemented here by alternating magneto-optic and non-chiral claddings of length *L*_G_ and *L*_N_, respectively, such that the accumulated modal phase difference Δφ = 2π (for first-order QPM) over the propagation distance (*L*_G_ + *L*_N_). Polarisation selectivity to eliminate feedback due to backward traveling TM modes in the laser can be provided by the selection rules of semiconductor quantum wells, which essentially makes the laser transparent to TM-polarised light at the emission wavelength. If necessary, a polarization filter can also be implemented^18^. In contrast, for MZIs to operate as TE-mode isolators, they require a full TE to TM RPC at the input, followed by a 1 × 2 coupler into the interferometer, where one or both of the waveguide branches have NRPS segments and/or reciprocal phase shifters (RPS). Next, a 2 × 1 coupler is used at the output where another full RPC^19, 20^ is needed. Each one of these components will have affiliated efficiencies and losses, so 1D 2-component FR isolators have great design appeal. Ring isolators will also need either two full RPCs, one on each end of a garnet top-cladded ring, or garnet could be coated inside the ring with a permanent magnet applying an out-of-plane field^21^. However, TE-mode ring isolators have not been demonstrated on SOI to date.

## Monolithically-Integrated Faraday Rotation Waveguide Isolators

**Figure 2:**
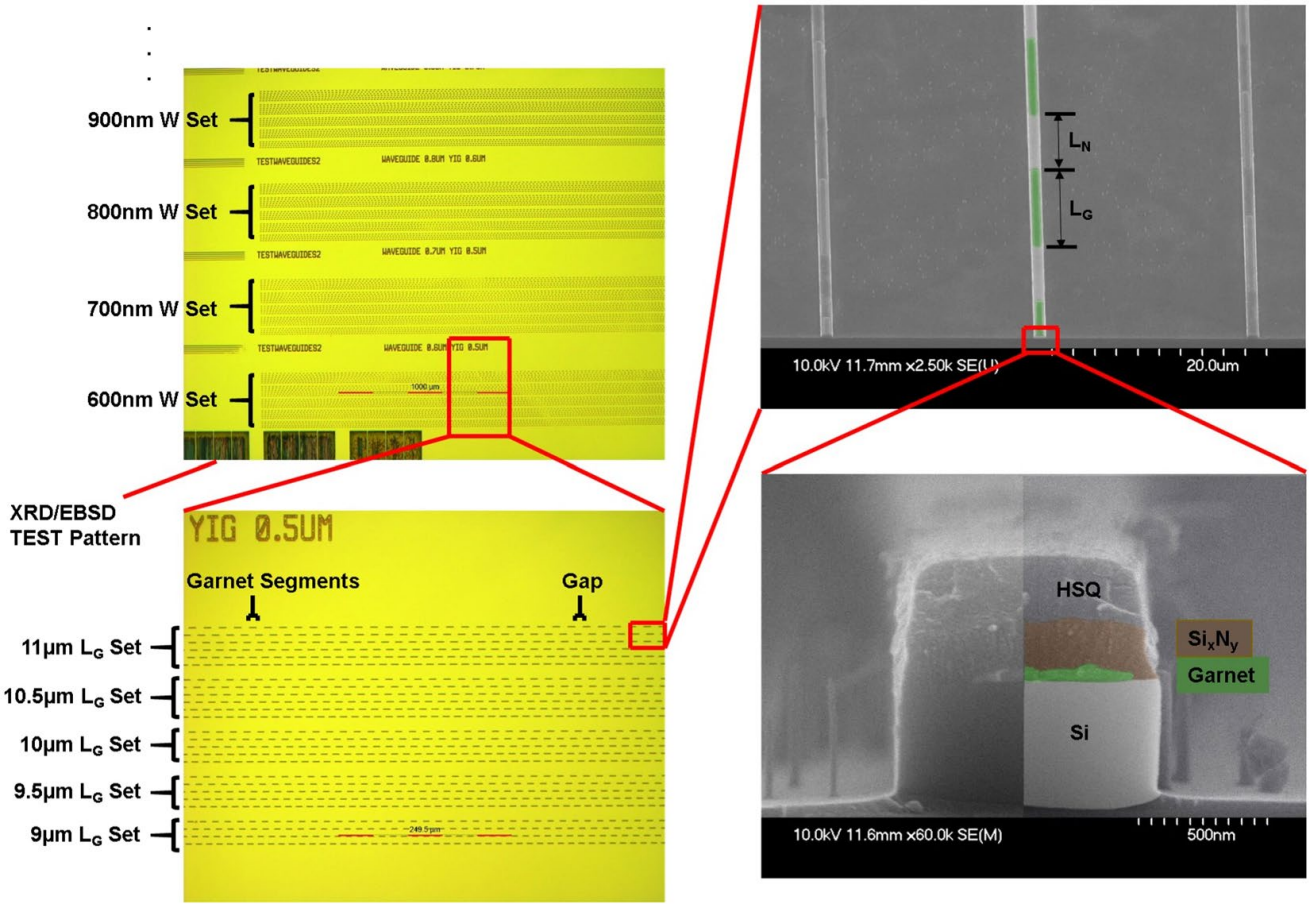
Monolithic integration of Faraday Rotation waveguide isolators. Optical (left) and scanning electron (right) micrographs showing the SOI waveguides with different widths (W), different garnet segment lengths (L_G_), and a cross section of Bi:TIG cladding on SOI waveguides with performance shown below.

The TE-mode SOI isolators introduced here used magneto-optical garnet claddings and evanescent coupling, Fig. 1a, similar to the NRPS devices, Fig. 1c. However, instead of inducing NRPS in TM-modes, waveguides were designed to allow propagation of two fundamental modes, TE- and TM-polarisation. Quasi-phase matching, Fig. 1b, was accomplished by periodic lift-off lithography^22, 23^ of the garnet cladding, and then by coating the waveguide with Si_x_N_y_ for approximate index matching to produce segments lengths corresponding to the characteristic beat-length (corresponding to Δφ = 2π), Fig. 2. For the garnet cladding, we initially used the most popular magneto-optical garnet, Ce-doped yttrium iron garnet (Ce:YIG), which was first integrated onto SOI by Bi *et al*.^3^ using pulsed laser deposition and an undoped YIG seedlayer annealed by rapid thermal annealing^24^. The highest Faraday Rotation (−3700 deg/cm) reported for SOI-integrated Ce:YIG uses a >45 nm YIG seedlayer^25^. However, the evanescent coupling between the propagating mode and its magneto-optical cladding exponentially decays with seedlayer thickness. Therefore, we are also reporting the first experimentally-verified devices that use a newly discovered seedlayer-free Bi-doped terbium iron garnet Bi:TIG^26^. The properties of the SOI waveguides and the cladding materials are presented in SI (section 2 and 3). As shown in Fig. 2, the garnet claddings are thin (<100 nm) but this is sufficient to have almost full interaction with the evanescent tail of the mode^22, 26^. As the net non-reciprocal mode conversion will depend on the modal overlaps with the magneto-optic garnet layer, this will substantively come from the garnet cladding coverage of the center portion of the waveguide, and hence there will be some degree of tolerance for the mask re-alignment step.

## Operating Principles

**Figure 3:**
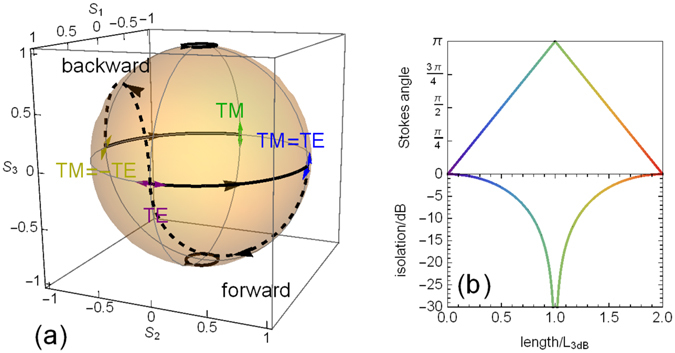
Operating principles of Faraday Rotation isolators. (**a**) Poincare sphere illustrating the polarization state evolution for the isolator device of Fig. 1a, where the solid lines correspond to nonreciprocal Faraday Rotation and the dashed lines to the half reciprocal phase conversion. (**b**) Relative Stokes vector angle and resulting isolation ratio obtainable vs element length, scaled to the length corresponding to 50% power transfer between modes, L_3dB_. The maximum degree of isolation corresponds to orthogonal Stokes vectors, or a relative angle of π.

In this paper, the Stokes parameters are used to represent the polarisation state of the light, which can be illustrated on the surface of the Poincare sphere as in Fig. 3a. Linear polarisation states correspond to the “equator”, and circular polarisation states to the “poles”. First, TE-polarised light is injected into the waveguide. The polarisation rotates to a linear TM = TE (+45°) state in the FR (solid trajectory) and then back into TE in the half RPC (dashed trajectory). Following the light propagation direction, back reflected light enters the isolator output where the polarisation state evolves from TE to TM = −TE (−45°) in the RPC and then to TM in the FR. These phase-matched trajectories can correspond to either non-birefringent or QPM waveguides. For an isolator, the backward output (TM-polarised) should ideally be orthogonal to the injected light (TE-polarised). This desired polarisation orthogonality is entirely attributable to the nonreciprocal FR.

Isolation by NRPS devices is typically reported as the difference between TM transmission in the forward and backward directions, which is usually inferred from the equivalent measurement of the difference in TM forward transmission when the transverse magnetic fields are reversed, Fig. 1c. This ‘equivalent’ measurement eliminates error caused by experimental variations in end-fire coupling at the two facets, and we employ the same characterisation technique in this paper. Therefore the effectiveness of FR for an isolator can be determined from the degree of orthogonality of the ouput polarisation states under reversal of the magnetisation. For example, the output of the FR element described in Fig. 3a would have orthogonal linear polarisation states under opposite magnetisations corresponding to ±π/2 (blue and yellow points) for TE-polarised input. Figure 3b shows the angle between the two output Stokes vectors as a function of element length (π corresponds to orthogonality) for phase-matched conversion, and the corresponding isolation ratio obtainable when combined with an ideal half RPC element. For a finite phase-mismatch, the angle between the Stokes vectors is constrained to values below the line in Fig. 3(b, top).

In order to obtain the isolation ratio of the integrated FR isolator, we note that the degree of orthogonality of the forward- and backward-propagation outputs can be obtained from the Stokes vectors, and will be unchanged with any reciprocal polarization conversion (since RPC can be represented as a rotation about an axis of the Poincare sphere). The angle between the two (normalized) Stokes vectors of opposite propagation directions *θ* is simply calculated from the scalar product, cos *θ* = ***S***_+_ · ***S***_−_. If a polarization selective element (in practice a combination of waveplate(s), i.e. reciprocal polarisation converter(s), and an ideal linear polarizer) is introduced to pass the ***S***_+_ polarization state, then Malus’ Law provides the relative transmission of the ***S***_−_ polarization state and hence the isolation ratio can be expressed,

1 $$\mathrm{Isolation}\,\mathrm{ratio}\,({\rm{dB}})=20\,{\mathrm{log}}_{10}|\cos \,\frac{\theta }{2}|=10\,{\mathrm{log}}_{10}\frac{1}{2}(1+{{\boldsymbol{S}}}_{+}\cdot {{\boldsymbol{S}}}_{-})$$

## Device Measurements

Using the Fabry-Perot technique, losses were measured in SOI waveguides with SiO_2_ upper claddings as 11.2 dB/cm and 14.7 dB/cm for the TE- and TM-polarised modes, respectively, which we attribute mainly to sidewall roughness. The QPM garnet-clad waveguides did not exhibit suitable Fabry-Perot fringes for loss measurements, but the average transmission was similar to SiO_2_ cladded guides for the TE-polarised mode and lower for the TM-polarised mode. The extra losses for the TM modes (which have a larger modal overlap with the cladding) were estimated as 5 dB/cm higher than the SiO_2_ cladded guides. Therefore, the 4.1 mm and 3.4 mm devices reported below have estimated forward waveguide losses of 4.6 and 3.8 dB, respectively.

**Figure 4:**
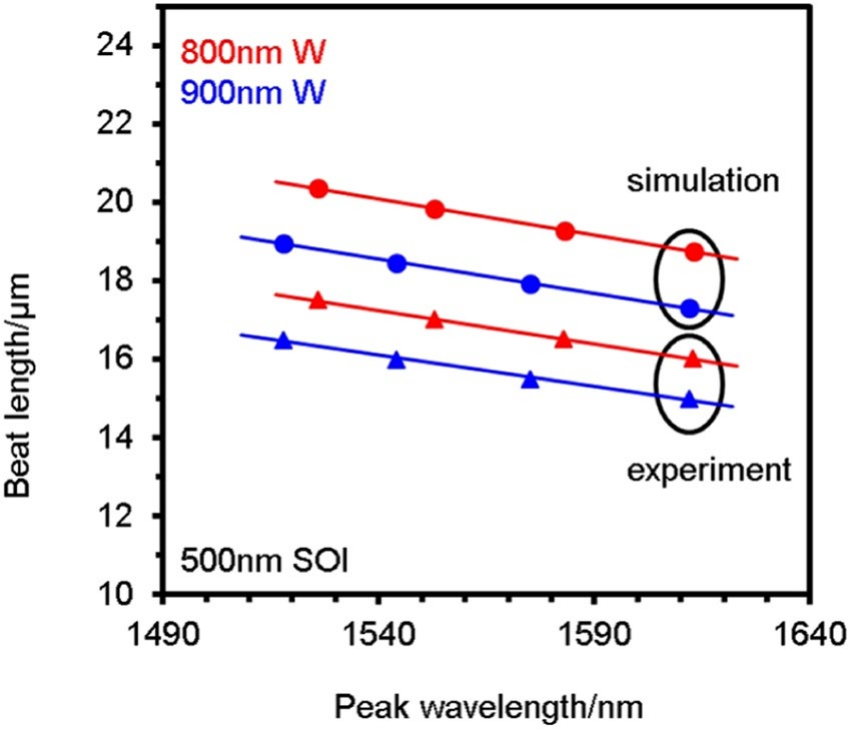
Simulation vs experiment determining optimal beat-lengths for isolation. Devices of 500 nm thick SOI, with the widths shown, experience modal birefringence such that Ce:YIG/YIG claddings require QPM with Si_x_N_y_. Optimal beat-lengths (see Fig. 1) were determined by simulation. Somewhat higher experimental birefringence shows that shorter beat-lengths were actually needed for optimal mode conversion. Every device was measured 3 times, corresponding to an uncertainty in measured wavelength <1 nm.

Our feasibility study involved 500 nm-thick SOI waveguides with top cladding segments of Ce:YIG on YIG overclad with silicon nitride and etched to a range of waveguide rib widths. The Ce:YIG/YIG cladding had thicknesses of 300 nm and 50 nm in open areas, which typically resulted in segment thicknesses of 80 nm and 15 nm after liftoff, respectively. Figure 4 shows the QPM beat-length required to achieve peak modal conversion as a function of wavelength for two nominal waveguide widths (800 and 900 nm). The experimental beat-lengths (*L*_G_ + *L*_N_) were consistently lower than the simulated beat-lengths, due to differences in the fabricated cross-sectional geometry from the simulated rectangular claddings, which leads to higher birefringence. However, the trends between the experiments and simulations had excellent agreement, which enables behavior predictions for the fabricated devices.

**Figure 5:**
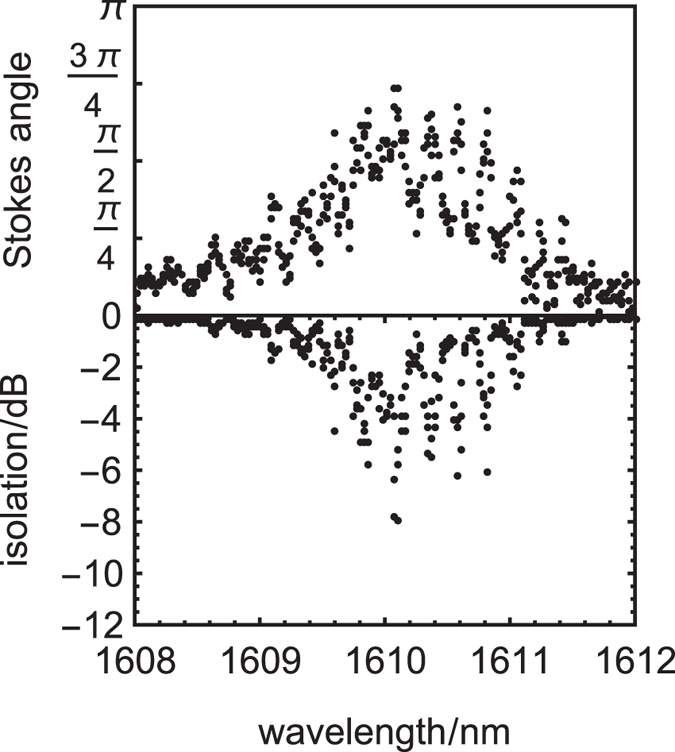
Performance of 500 nm SOI FR isolator with Ce:YIG/YIG claddings. (top) The Stokes vector angles of opposite magnetic saturation, where a peak relative angle of ~3/4 π was observed with this 4.1 mm long device. The oscillations are Fabry-Perot fringes, which can be avoided with anti-reflection facets. (bottom) Calculated isolation ratios using Stokes angle data and an idealised waveplate for reciprocal polarisation conversion.

These first FR isolators exhibited shape-induced birefringence (SOI core: 500 nm × 900 nm) and they were cladded with high FR Ce:YIG (−3700 deg/cm) on an undoped YIG seedlayer^25^. The Stokes parameters at the output of these 4.1 mm long devices were measured with forward and backward magnetic saturation, Fig. 5a. Enhanced FR at Fabry-Perot resonances at the Δλ free spectral range (FSR) led to the oscillations observed, but these would be avoided in integrated isolators via anti-reflection facets. A maximum Stokes vector angle of nearly 3/4π (0.74π) was observed for a 4.1 mm long device when phase-matching was achieved. An ideal (1 ± 0.0027)π could be achieved with a longer device length of 5.5 mm although losses would increase to 6 dB. The QPM period of the device with the performance shown in Fig. 5 was 15 μm, which is a resolution easily achieved with standard photolithography. Importantly, even with a Stokes vector angle of 0.74π, an isolation ratio of −8 dB could be obtained using a conventional waveplate for the half reciprocal polarization rotator (see Figs 1 and 3). Integrated half RPCs have been achieved with specific lengths of birefringent waveguides that have asymmetric profiles^27–29^.

**Figure 6:**
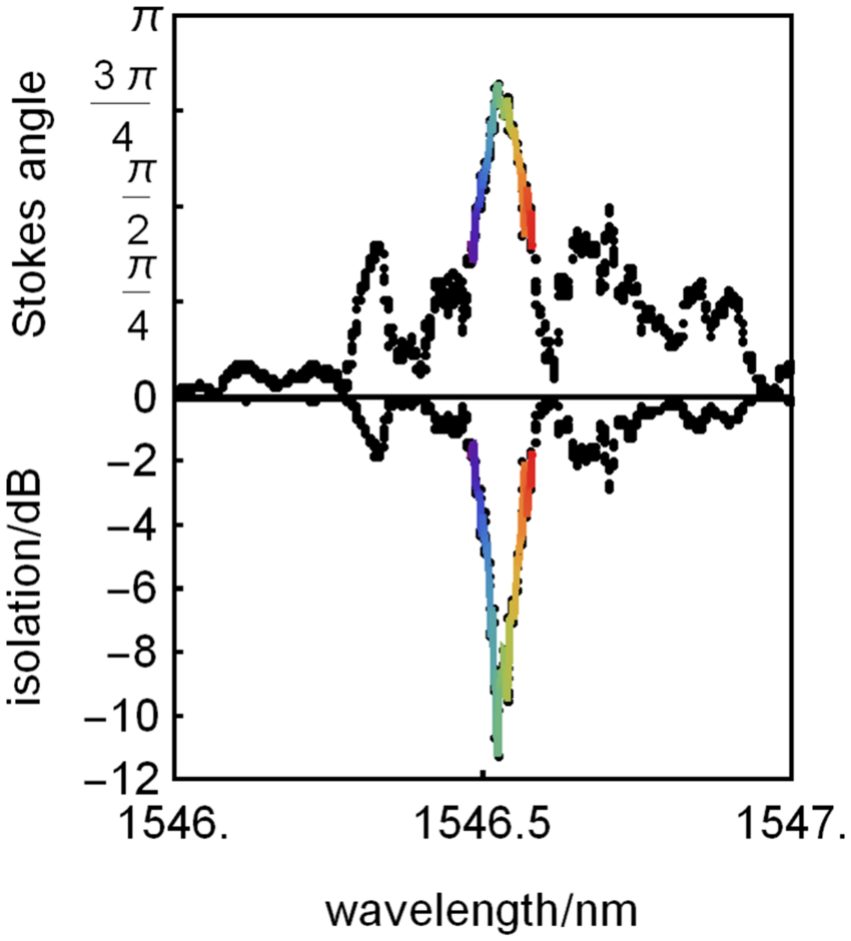
Performance of 340 nm SOI FR isolator with seedlayer-free Bi:TIG claddings. (top) The Stokes vector angle of opposite magnetic saturation, where a peak relative angle of 0.83π is observed. (bottom) Calculated isolation ratios using the data in (**a**) and an idealised waveplate for reciprocal polarisation conversion.

The next FR isolator designs had thinner SOI (340 nm thick Si) to enhance the modal interaction with the garnet cladding, which here was seedlayer-free Bi:TIG^26^ (80 nm thick), Fig. 2, together with Si_x_N_y_ for QPM. Here, the mode conversion peak was narrower in wavelength and the Fabry-Perot resonances were not as pronounced. The maximum Stokes vector angle was 0.83π for a 3.4 mm long device even with the non-ideal cross section (shown in Fig. 2). An ideal (1 ± 0.0014)π could be achieved with a longer device length of 4.1 mm although losses would increase to 4.6 dB. The QPM period of the device whose performance is shown in Fig. 6 was 8 μm (SOI core: 340 nm × 900 nm), which is still a resolution that is easily achieved with standard photolithography. Importantly, with a Stokes vector angle of 0.83π, we deduce a maximum isolation ratio of −11 dB, which is similar to ratios obtained with other first reports of new TM-mode isolator designs^1, 5, 6, 30^.

## Conclusion

This is the first report of an on-chip integrated TE-mode FR SOI isolator, and therefore is an important step in achieving new applications for photonic integrated circuits. These FR isolators are simple 1D (all in-line) 2-element waveguides with unique QPM claddings to overcome waveguide birefringence. Although this paper focused on TE-modes as a thus far unobtainable but necessary feat, the fundamental phenomenon of nonreciprocal mode conversion can also be implemented to provide TM-mode isolation. Compared to NRPS devices, the design is sublimely simple, and the garnet claddings can be saturated at low and monodirectional applied magnetic field due to the longitudinal nature of FR which corresponds to the preferred in-plane orientation for the garnet shape anisotropy. The devices presented here are also the only nonreciprocal devices to have used only lithography with sputter deposition, which are workhorse processes in the semiconductor, hard drive, and photonics industries. This is a significant improvement in potential for commercial upscaling compared to the other garnet-integration methods of pulsed laser deposition and wafer bonding.

SOI (500 nm Si) isolators were first tested with high-FR Ce:YIG to achieve rotations of 0.74π, which could lead to isolation ratios of −8 dB for a 4.1 mm long device (900 nm wide) if combined with an appropriate reciprocal polarization convertor which was simulated here as an idealised waveplate. Thinner SOI (340 nm) was then used for better modal interaction with seedlayer-free Bi:TIG to achieve rotations of 0.83π, corresponding to −11 dB for a 3.4 mm long device (900 nm wide). The beat-length of this device was 8 μm, so an ideal device (1 ± 0.0014π, capable of providing over 30 dB of isolation) could be achieved by increasing the device length to 4.1 mm with losses of only 6 dB. The inline geometry of the FR isolator avoids additional losses at splitter and couplers, along with the potential of lithography to provide tapered transitions between segments/elements to avoid the sharp impedance discontinuity with bonding techniques, suggests that FR isolators could have lower overall losses than the NRPS schemes.

## Methods

### Material and Device Fabrication

Supplementary Fig. S1 shows the device fabrication process for 1D non-reciprocal isolators on SOI substrates. First, a bi-layer PMMA (4% 2041, 15% 2010) lift-off mask^22, 23^ was prepared for garnet segments on a bare Si (500 nm/340 nm)/SiO_2_ (3 µm/2 µm)/Si SOI wafer, and the QPM pattern was written by electron-beam lithography. Then Ce:YIG/YIG(80 nm/15 nm) or Bi:TIG(75–80 nm) thin film were deposited by rf sputtering, followed by lift-off in hot acetone (50 °C) and cyrstallization by RTA (rapid thermal annealing) at 900 °C for 2 minutes in O_2_ at 120 mBar (details in supplemental information, section 2). 100 nm-thick Si_x_N_y_ was then deposited over the sample by ICP CVD (Oxford Instrument). Afterwards, a 650 nm thick hydrogen silsesquioxane (HSQ) resist was spin-coated above the Si_x_N_y_ layer. The waveguide pattern with widths ranging from 600 nm to 1100 nm was defined by electron-beam lithography and developed in tetramethylammonium hydroxide (TMAH). The patterns were subsequently transferred to the Si_x_N_y_ by reactive ion etching (80 + RIE, Oxford Instrument) in CHF_3_/O_2_ and then to the Si layer by STS ICP (SPTS) in SF_6_/C_4_F_8_ gas.

### Optical isolation characterization

In order to characterize the isolators shown in Fig. 2, both edges of the samples were cleaved to a convenient device length for a Fabry-Perot (FP) cavity. The set-up for the transmission measurement is presented in Fig. S2. Continuous-wave tunable laser light (TE-polarised) was injected into the guides using end-fire coupling, and the output was passed through a polarising beam-splitting cube to separate the TE- and TM-polarised components. On scanning the wavelength (1500–1630 nm), the polarisation mode conversion, *P*_TM_/(*P*_TE_ + *P*_TM_), exhibited a peak when the phase-matching criterion was satisfied. The output polarisation states for candidate mode conversion peaks were then fully characterized using a Thorlabs© PAX5710 polarimeter in place of the combination of photodetector and lock-in amplifier to obtain the output Stokes parameters as a function of wavelength.

Similar to MZI and ring resonator measurements without fiber pigtail packaging, the direction of light propagation was fixed and the longitudinal magnetisation was set in either forward or backward direction with an external 1.2 kOe magnetic field. This produced the same permittivity tensor as forward and backward propagating beams without changing coupling, thereby minimizing measurement error. This magnetic field was sufficient to saturate the ferromagnetic garnet film and optical characterizations were performed using the remanent magnetization.

### Data Availability

The datasets generated and analysed during the current study are available in the University of Glasgow Enlighten Research Data repository, http://dx.doi.org/10.5525/gla.researchdata.397.

## Electronic supplementary material

Supplemental Information
