# Supplementary material for: Monolithically-Integrated TE-mode 1D Silicon-on-Insulator Isolators using Seedlayer-Free Garnet
Source: Sci Rep. 2017 Jul 19;7:5820. doi: 10.1038/s41598-017-06043-z (PMC5517653; doi:10.1038/s41598-017-06043-z)
Supplement: Supplementary file 1 — Supplemental Information [file 41598_2017_6043_MOESM1_ESM.pdf]

Monolithically-Integrated TE-mode 1D Silicon-on-Insulator Isolators using Seedlayer-Free Garnet

Cui Zhang,[1†] Prabesh Dulal,[2†] Bethanie J. H. Stadler, [2,3*] David C. Hutchings[1*]

[†] Equal contributions
* Corresponding authors: stadler@umn.edu, David.Hutchings@glasgow.ac.uk
[1] School of Engineering, University of Glasgow, Glasgow G12 8LS, United Kingdom
[2] Chemical Engineering and Materials Science and [3]Electrical and Computer Engineering, University of Minnesota, Minneapolis, Minnesota 55455, United States.

Supplemental Information

1. Device Fabrication and Characterisation Process

Figure S1 presents the fabrication process flow of 1D isolator on SOI platform as described in the methods part of the manuscript. The left part shows the first stage after periodic garnet segments, either Ce:YIG/YIG or Bi:TIG, radio frequency (RF) deposition, lift-off, and Rapid Thermal Annealing (RTA). The right part shows the finished device after Si_xN_y deposition, waveguide defined by e-beam lithography and plasma processing. Although electron beam lithography was used for both lithography stages, the principal advantage of this is to facilitate the re-registration of the mask writing, rather than resolution requirements.

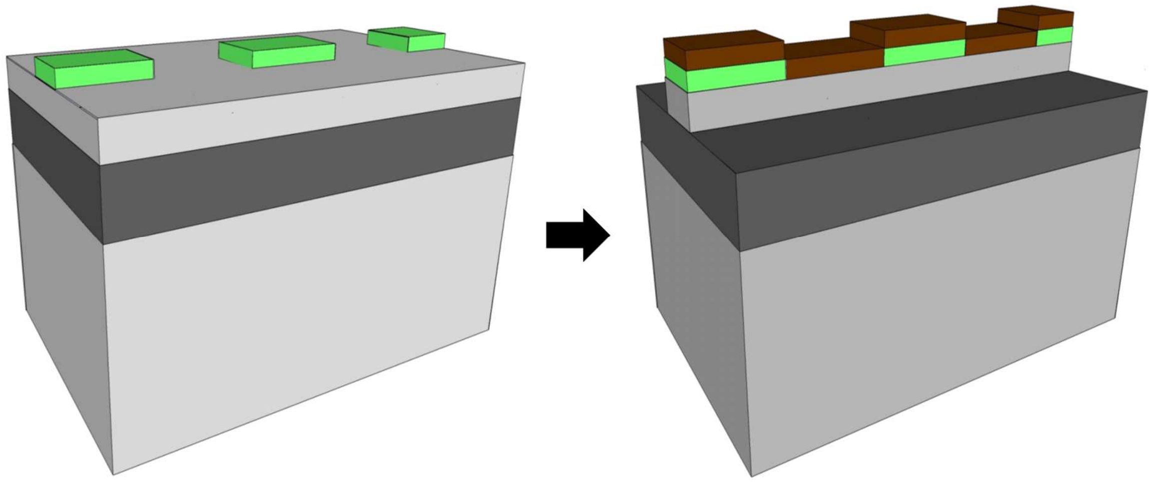

Figure S1. Schematics illustrating the device fabrication process.

Figure S2 presents the setup for transmission and isolation measurements as described in the methods part of the manuscript. The cleaved sample was saturated longitudinally along the light propagation direction by placing it between the poles of a permanent yoke magnet, of about 1.2 kOe, before measurements. The sample was removed from the magnet and only remanent magnetisation remained for the measurement process.

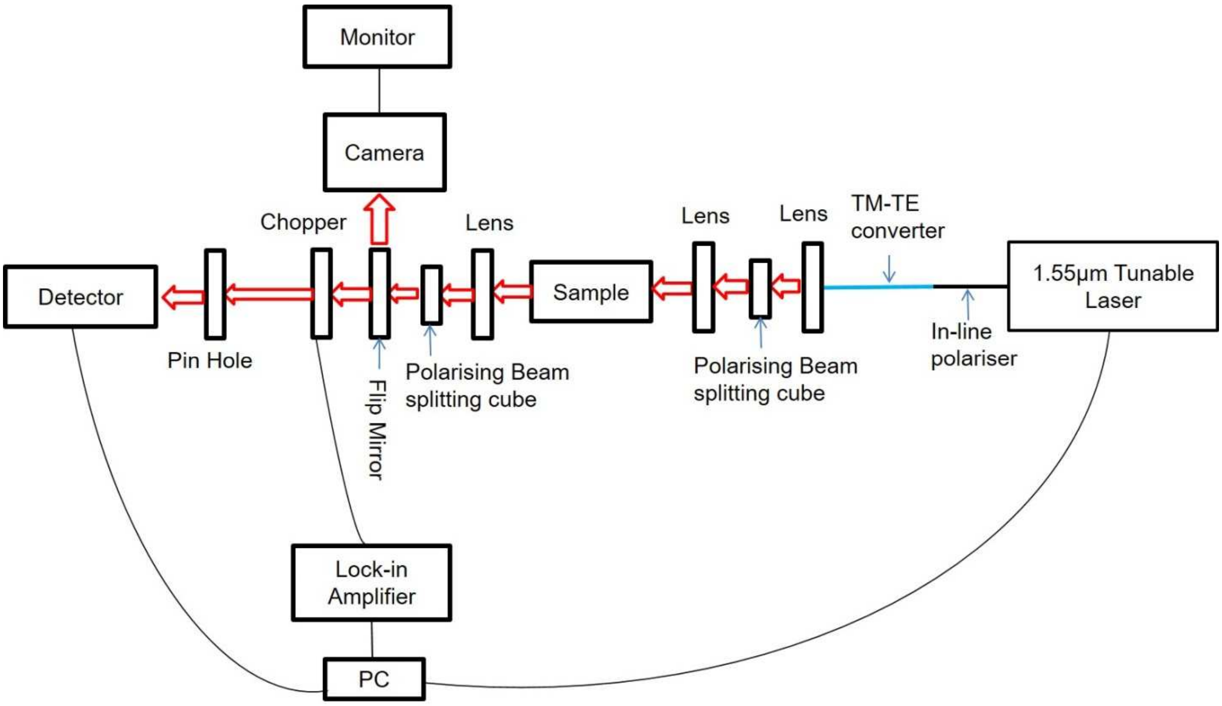

Figure S2. Transmission measurements rig set-up.

2. Deposition and characterization of Ce:YIG/YIG and Bi:TIG

Ce:YIG and Bi:TIG films for waveguide claddings were grown using RF reactive sputter deposition with an Ar/O_2 plasma followed by rapid thermal anneal. Prior to the deposition of Ce:YIG, a 50 nm seedlayer of YIG was grown using a composite FeY target that was sputtered at 240 W, flowing in Ar at 20 SCCM and O_2 at 2.0 SCCM. This amorphous film was then annealed (RTA) at 900°C for 2 mins in an O_2 atmosphere which led to the formation of the crystalline YIG phase confirmed by XRD. Following this, the Ce:YIG film was grown on the YIG seedlayer by sputtering the composite FeY target at 240 W and a Ce target at 40 W, flowing in Ar at 20 SCCM and O_2 at 2.0 SCCM. The resulting film was again annealed (RTA) at 900°C for 2 mins in an O_2 atmosphere which led to the formation of single phase Ce:YIG, also confirmed by XRD. For the deposition of Bi:TIG, an Fe target was sputtered at 220 W and individual Tb and Bi targets were sputtered at 110 W and 10 W respectively. The deposition was performed with 20.4 SCCM Ar and

2.0 SCCM O_2 flow. This was followed by RTA at 900°C for 2 mins in an O_2 atmosphere, which resulted in single phase Bi:TIG as confirmed by XRD.

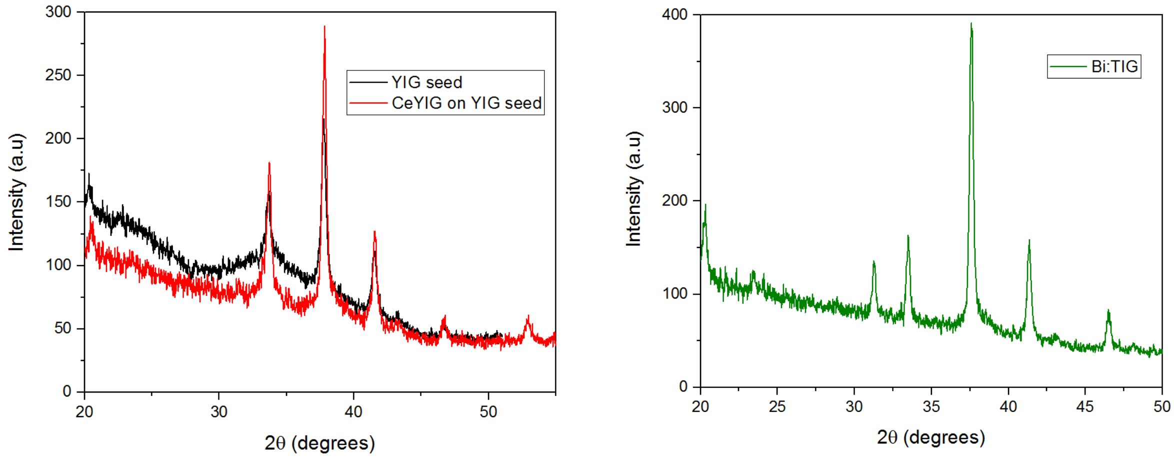

Figure S3. XRD measurements of the deposited garnet films

3. Comparisons with previous work

Reference, type, year	Input Pol.	Fabrication	Size/mm^2	Isolation/dB	Loss/dB
3. MRR (2011)	TM	PLD+anneal	0.3 × 0.05	19	20
4. MZI (2014)	TM	Direct bonding	1.5 × 0.5	27	13.4
5. MZI (2012)	TM	BCB bonding	1.5 × 0.004	11	14
6. MRR (2011)	TM	Direct bonding	2 × 2	9	
7. MRR (2016)	TM	Direct bonding	0.6 × 0.6	32	10
19. MZI (2016)	TE	Direct bonding	2.5 × 1.5	26.7	18.5
20. MZI (2013)	TE	BCB bonding	6 × 0.2	32	22
30. MZI (2012)	TM	Direct bonding	1.5 × 1.5	18	21
CeYIG/YIG FR (this work)	TE	Sputter+RTA	4.1 × 0.001	8.0	3.8
BiTIG FR (this work)	TE	Sputter+RTA	3.4 × 0.001	11.6	4.6

Table S1. Comparison with selected SOI integrated isolator studies. Types are MRR: micro-ring resonator, MZI: Mach-Zehnder interferometer, FR: Faraday rotation.

Table S1 show a comparison of the current Faraday rotation study with previous selected SOI integrated isolator studies based on nonreciprocal phase shifters. Device sizes may be approximate, and the insertion loss value generally attempts to discount the facet coupling losses from chip to air or fiber.

4. Analysis

Faraday rotation is manifest as nonzero imaginary elements in the permittivity tensor for a magneto-optic medium (e.g. iron garnet). For a longitudinal magnetization parallel to the *z*-axis:

$$\varepsilon = \begin{bmatrix} \varepsilon_{xx} & \mathrm{i}\varepsilon_{xy} & 0 \\ -\mathrm{i}\varepsilon_{xy} & \varepsilon_{yy} & 0 \\ 0 & 0 & \varepsilon_{zz} \end{bmatrix} \quad \text{(s-1)}$$

In the non-birefringent case, the modes correspond to left- and right-circular polarisations which propagate with different phase velocities. Injection of linear polarized light leads to mode-beating and manifests itself as a rotation of the plane of polarization, i.e. Faraday rotation, with a rotation per unit distance given by,

$$\frac{\theta_F}{L} = \frac{\pi \varepsilon_{xy}}{n\lambda} \quad \text{(s-2)}$$

where $n = \sqrt{\varepsilon_{ii}}$ is the refractive index. The key function for an optical isolator is that the throughputs (corresponding to opposite magnetisation with respect to the propagation direction) are orthogonally polarized with respect to each other after a specific distance (45° rotation of the polarization plane), and consequently can be either passed or rejected with an appropriate polarization selective element. This orthogonality requirement also applies to quasi-phase-matched nonreciprocal polarization mode conversion, and is also unchanged if any reciprocal polarization mode conversion occurs.

Figure S4 (left) shows the measured Stokes parameters for the Bi:TIG clad SOI waveguide over the indicated wavelength range in figure 6 with the large and small points representing opposite magnetization. In the integrated isolator application, polarization selectivity is normally constrained to TE- or TM-polarised modes (as with the TE-polarisation selection rules for a quantum well laser discussed earlier), and hence additional reciprocal polarization conversion is normally required. In our analysis we assume that this is accomplished with an idealized waveplate, such that the forward propagating light is completely converted to TE-polarisation ***S***=(1,0,0). It is clear from Figure S4 (left) that the Stokes parameter trajectories around the peak nonreciprocal mode conversion do not follow a

simple trajectory on the Poincare sphere. We attribute the complicated trajectories to the slight offset of the garnet cladding from the waveguide rib center. This offset could lead to additional reciprocal polarisation mode conversion and/or a longitudinal variation in the phase-matching criteria. Figure S4 (right) shows the corresponding Stokes parameters for the backward-propagating light upon the required transformation. We anticipate that this reciprocal polarization converter could be a bespoke asymmetric-profile waveguide[27–29], or the integrated polarization aligner device based on universal 3 dB polarization converters and differential phase shift elements[16]. The calculated reciprocal transformation is obtained using a Mueller matrix approach. A perfect isolator would show a backward-propagating polarization state corresponding to TM-polarisation, ***S***=(-1,0,0). The green points show the maximal degree of nonreciprocal polarisation conversion for this sample, and appear to correspond to a somewhat shorter sample length than ideal, as discussed in the main text.

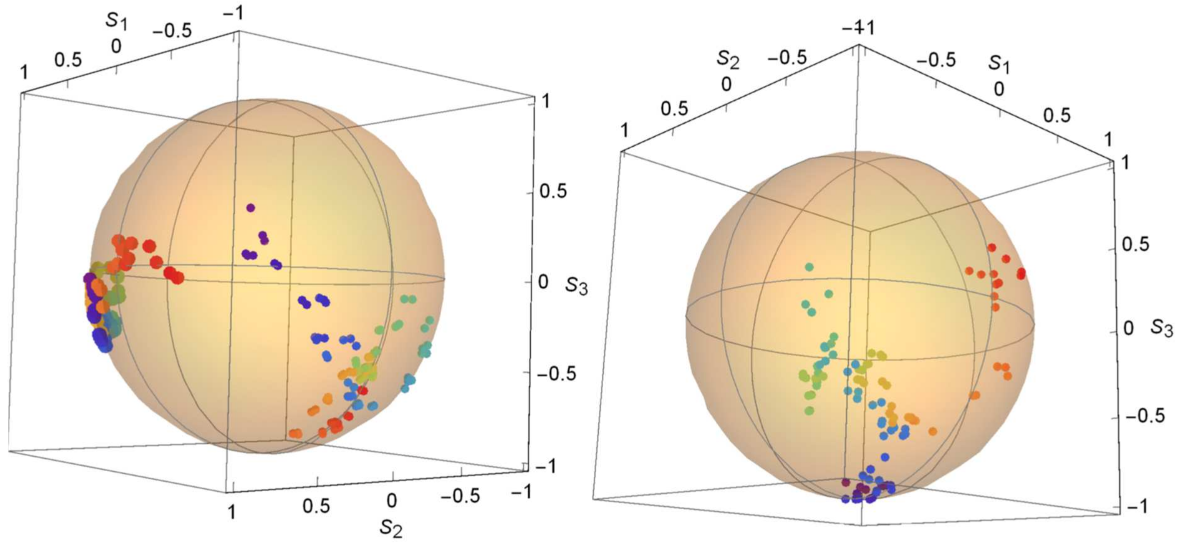

Figure S4. Poincare sphere representations of the output polarisation state. (left) shows the raw, measured Stokes parameters for the Bi:TIG clad SOI waveguide corresponding to figure 6 with the large and small points representing opposite magnetisation and the colour matches the appropriate wavelength across the QPM conversion peak from Fig. 6. **(right)** shows the Stokes parameters which would be obtained for backward-propagating light if an idealised reciprocal polarisation converter adjusted the forward-travelling light to TE-polarisation.
